# Changes in the composition of urine over six hours using urine dipstick analysis and automated microscopy

**DOI:** 10.1186/s12882-024-03933-z

**Published:** 2025-01-08

**Authors:** Pranav C. Parikh, Serena D. Souza, Wassim Obeid

**Affiliations:** 1 Gilman School, 5407 Roland Ave, Baltimore, MD 21093 USA; 2 Division of Nephrology, Johns Hopkins School of Medicine, 1830 E Monument Street, Room 416, Baltimore, MD 21287 USA

**Keywords:** Sediment, Kidney disease, Pathological Casts, Urine Analysis, Urine Microscopy, Automated Microscopy, Dipstick Analysis

## Abstract

**Background:**

Urinalysis is a commonly performed test for the diagnosis and prognosis of kidney disease in hospitalized patients. It involves examining the chemical composition of the urine and microscopy to examine the cells and casts. In clinical settings, urinalysis is frequently delayed by several hours after sample collection and held at room temperature. The purpose of this study is to investigate the changes in urine composition over set time intervals to confirm the reliability of urinalysis when there are delays in performing the tests.

**Methods:**

We obtained 15 mL of urine from the Foley catheters of five patients in the intensive care unit. We utilized the state-of-the-art IDEXX SediVue Dx ® machine to perform urine microscopy and the Siemens CLINITEK Status + Urine Analyzer to perform the dipstick tests. We performed microscopy and dipstick tests at 0, 1, 2, 4, and 6 h. Between the two testing methods, 30 individual components were tested in the urine. We calculated the %CV for each component by taking four repeated measurements at one time period for multiple samples.

**Results:**

After calculating the %CV for each component, we analyzed the trend for each constituent over the 6 h. If the percent change over the six-hour interval was ± twofold than the %CV, we determined time to influence the results. Significant changes were seen in bacteria as the levels increased, red blood cells and pathological casts where the level decreased, and crystal levels were determined inconclusive due to fluctuations in the results. All other components were found to remain unchanged.

**Conclusions:**

Timely urine analysis is necessary for accurate results as delayed analysis can considerably change the makeup of urine, which can affect clinical decisions and patient management.

**Supplementary Information:**

The online version contains supplementary material available at 10.1186/s12882-024-03933-z.

## Introduction

Urine Analysis (UA) is a cornerstone diagnostic medicine test used to detect and monitor medical conditions ranging from infections, metabolic disorders, malignancies, and other kidney diseases [1, 2]. As one of the oldest medical tests, UA is usually conducted by the clinicians themselves at the bedside or in a microscopy room near the patient units [3]. The performance of this test is inconsistently delayed by several hours after the urine sample is collected due to competing clinical priorities [4].

UA involves testing the chemical composition of the urine and microscopy to determine the cells and casts. Although urine microscopy is performed manually in clinical settings, automated instruments have become increasingly used to perform this task. For example, the IDEXX SediVue Dx ® was developed for digital urine microscopic examination [5]. Historically, it has primarily been only used for veterinary testing and studies, but its function allows for more than satisfactory human urine measurements [6].

This study aims to investigate the changes in urine composition at room temperature over six hours to confirm the reliability of urine microscopy findings when the tests are delayed. We stored the samples at room temperature between the readings to mimic clinical settings in which urine samples are stored in microscopy rooms or kept in laboratory coat pockets. We utilized the automated urinalysis dipstick tests and state-of-the-art IDEXX SediVue Dx ® machine to perform urine microscopy and sediment analysis for accurate measurements [2]. We hypothesize that components such as bacteria will increase because their proliferation through binary fission happens rapidly. In contrast, other components, such as red and white blood cells, will reduce over time due to degradation and cell lysis.

Similar experiments have been performed with animal urine samples using the IDEXX SediVue Dx ® machine, however, none have tested human urine [7]. Although prior studies have investigated changes in urine composition using manual observations, none have performed these investigations using automated and digitalized methods where inter-observer reliability is minimized [8]. This study meticulously examines the changes over four-time intervals. Such insights could potentially have valuable implications in a clinical context with patient management as it would reduce diagnostic errors.

## Methods

From the nephrology consultative service in the ICU, we collected urine samples into sterile urine cups from 5 patients with Foley catheters using aseptic techniques. This ensured a clean and standardized sample collection method which minimized contamination risk to maintain sample integrity. After collection, the samples were promptly transported to the laboratory for clinical examination and analysis.

Dipstick urinalysis was performed on each urine sample using reagent strips (SIEMENS Multistix® 10 SG) to detect various chemical components in urine. The strips were immersed in the urine samples for 3–5 s, allowing the reagents to react with the urine. After removing excess urine by dabbing the sides of the dipstick, the strips were placed into the Siemens CLINITEK Status® + Analyzer for automated reading and interpretation of the results.

**Fig. 1:**
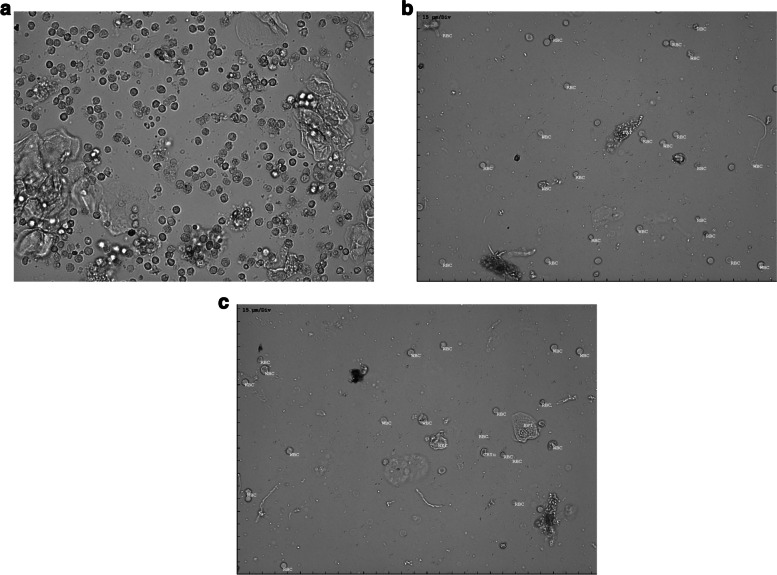
A Representative Example of the IDEXX SediVue Dx ® Urine Microscopy Images (Unlabeled and Labeled). **a**: Unlabeled Image. **b**: An image with labels of RBC, WBC, and Pathological Cast. **c**: An image with labels of RBC, WBC, Crystal, and Epithelial Cell

Microscopic examination was conducted using the IDEXX SediVue Dx ® instrument. This advanced instrument combines automated urine chemistry analysis with digital microscopy, with over 70 images, providing highly accurate results [3]. A precise volume (165 µL) of each urine sample was pipetted into the instrument's cartridge. The SediVue Dx ® then performed a comprehensive analysis, encompassing the identification and quantification of cellular elements, casts, crystals, and other particulate matter in the urine [9]. (Fig. 1a-c).

To capture the changes in urine composition, these variables were analyzed at specific four time points: 1 h, 2 h, 4 h, and 6 h after the initial collection. The samples were left at room temperature between measurements.

## Statistical analysis

We calculated the percentage coefficient of variation (%CV), for each component by taking four repeated measurements at Hour 6 of samples 1, 3, and 5. We used the average %CV for each element to determine whether the change was within the margin of error or an actual meaningful change. As the sample size was small, we studied the components that had %CV of ≤ 20%. If the percent change over the six-hour interval was over ± twofold of the average %CV, we determined time to influence the results. On the other hand, if the percent change fell within the range of the ± twofold of average %CV, the component remained unchanged over the period.

## Results

The values of the five individual samples at baseline (Hour 0) are depicted in Table 1. Most components assessed by microscopy and dipstick analysis were detectable, enabling trend evaluation. The average %CV for each component is shown in Supplementary Table 1. Components that exhibited %CV within the acceptable range (< 20%) were analyzed across the five time points.

**Table 1:** Data for All Components at Hour 0 for the Five Samples

IDEXX SediVue Dx ® Data					
**Particle/HPF**	Sample 1	Sample 2	Sample 3	Sample 4	Sample 5
RBC (Red Blood Cell)	34.46	20.12	28.59	3.15	290.1
WBC (White Blood Cell)	6.43	6.19	277.76	1.02	33.71
CRY (Crystals)	0.17	0.88	0.81	0.04	0.31
CaOxd (Calcium Oxalate)	0	0	0	0	0
TRI (Triple Phosphate Crystals)	0	0	0	0	0
CRYu (Unspecified Crystals)	0.17	0.88	0.81	0.04	0.31
AMB (Amorphous Crystals)	0	0	0	0	0
BIL (Bilirubin)	0	0	0	0	0
HYA (Hyaline Casts)	0	0.03	0.02	0	0.02
PAT (Pathological Casts)	0.28	0.11	0.02	1.67	0
NEC (Necrotic Material)	0.19	1.88	3.32	0.11	0.55
EPI (Epithelial Cells)	0	0.03	2.36	0.02	0.05
YEA (Yeast)	0	0	0	0	0
BACr (Rod-shaped Bacteria)	9.04	2.54	3.54	3.93	4.6
BACc (Cocci-shaped Bacteria)	32.23	7.98	7.22	41.37	4.05
BACcs (Bacteria, cocci in clusters)	31.18	7.98	6.09	40.43	1.52
BACcc (Bacteria, cocci in chains)	1.05	0	1.14	0.93	2.52
MUC (Mucus)	66.96	102.29	10.84	31.57	150.74
SPRM (Spermatozoa)	0.02	0	0	0.04	0
CRYd (Crystals, drug-induced)	0.04	0.54	0.06	2.7	5.88
**Siemens CLINITEK Status® + Analyzer Data**					
GLU (mg/dL) (Glucose)	Negative	100	Negative	Negative	Negative
BIL (Bilirubin)	Negative	Large	Negative	Negative	Small
KET (mg/dL) (Ketones)	Negative	Negative	Negative	Negative	15
SG (Specific Gravity)	1.02	1.02	1.02	1.02	1.03
BLO (Blood)	Large	Large	Large	Moderate	Large
pH	5.5	7	6	5.5	7
PRO (mg/dL) (Protien)	100	300	100	300	100
URO (E.U./dL) (Urobilinogen)	1	1	0.2	0.2	1
NIT (Nitrites)	Negative	Negative	Negative	Negative	Negative
LEU (Leukocytes)	Negative	Trace	Large	Negative	Negative

### Changes in components of automated microscopy

Among the components examined by the IDEXX Sedivue Dx ® machine examined, cocci-shaped bacteria (BACc), red blood cells (RBC), pathological casts (PAT), and crystals (CRY/CRYu) had changed significantly out of the range of the %CV. White blood cells (WBC) decreased 7.71%, but the %CV was 9.94%, suggesting that the count remained stable through this time frame.

The BACc count showed a notable increase over 6 h, with a percent change of 20.95%, exceeding its %CV of 2.8 (Table 2, Supplementary Fig. 1). This suggests rapid bacterial growth in urine samples at room temperature.

**Table 2:** Change in Cocci-shaped Bacterial Counts (BACc) over time

Cocci-shaped Bacterial Counts (BACc Particles/HPF)					
Sample	Hour 0	Hour 1	Hour 2	Hour 4	Hour 6
1	32.23	35.76	34.78	35.36	48.24
2	7.98	7.8	7.47	7.08	6.61
3	7.22	6.22	6.64	6.43	6.45
4	41.37	26.26	47.76	40.62	47.98
5	4.05	4.1	5.49	3.48	3.02
Average	18.57	16.03	20.38	18.59	22.46
% Change Hour 0 to Hour 6	20.95				

In contrast, the levels of RBC (Table 3 and Supplementary Fig. 1) and PAT (Table 4 and Supplementary Fig. 1) exhibited a decrease over time. For evaluating RBC trends, one sample was excluded as it had visible blood in the urine at baseline. The percent decrease in RBCs over 6 h was 28.46%, surpassing its %CV of 5.54. Three samples initially had PAT, and two showed a continuous decline, while one displayed a modest decrease until hour 4. Overall, a decrease in PAT content was observed.

**Table 3:** Change in Red Blood Cell (RBC) counts over time

Red Blood Cell Counts (RBC Particles/HPF)					
Sample	Hour 0	Hour 1	Hour 2	Hour 4	Hour 6
1	34.46	28.69	23.16	19.76	12.51
2	20.12	18.16	19.29	20.88	21.92
3	28.59	23.89	26.98	28.95	25.61
4	3.15	2.59	2.53	1.82	1.71
5	290.1	324.78	259.39	337.35	297.5
Average^a^	21.58	18.35	17.99	17.85	15.485
% Change Hour 0 to Hour 6^a^	−28.46				

^a^Sample 5 was excluded from Average calculations as the urine contained high amounts of blood. A negative sign suggests that there was a decrease over time

**Table 4:** Change in Pathological Cast Counts (PAT) over time

Pathological Cast Counts (PAT Particles/HPF)					
Sample	Hour 0	Hour 1	Hour 2	Hour 4	Hour 6
1	0.28	0.11	0.17	0.26	0.04
2	0.11	0.07	0.06	0.03	0.12
3	0.02	0.02	0	0	0.02
4	1.67	1.14	1.26	1.33	0.88
5	0	0.02	0.11	0.02	0.05
Average^a^	0.68	0.44	0.49	0.54	0.34
% Change Hour 0 to Hour 6^a^	−50.00				

^a^Samples 3 and 5 are not included from Average calculations due to non-detectable values of the casts. A negative sign suggests that there was a decrease over time

The levels of CRY/ CRYu (Table 5 and Supplementary Fig. 1) fluctuated throughout the 6 h, with the percent change sometimes exceeding and sometimes within the %CV range. Notably, in sample 1, the count jumped to 8.12 p/HPF at hour 6, despite being t < 1 p/HPF for the first 4 time points. Due to these inconsistent results, we could not draw definitive conclusions about the influence of time on crystal levels.

**Table 5:** Change in Crystal Counts (CRY/CRYu) over time

Crystal Counts (CRY and CRYu Particle/HPF)					
Sample	Hour 0	Hour 1	Hour 2	Hour 4	Hour 6
1	0.17	0.32	0.26	0.88	8.12
2	0.88	0.61	0.4	0.6	0.98
3	0.81	0.09	0.11	0.02	0.02
4	0.04	0.09	0.11	0.26	0.17
5	0.31	0.27	0.31	0.37	0.3
Average^a^	0.51	0.27	0.23	0.31	0.37
%change over Hour 0 to Hour 6^a^	−27.94				

^a^Sample 1 was excluded from Average calculations due to an extreme outlier at the Hour 6 time point

### Changes in components of dipstick analysis

Dipstick urinalysis also revealed changes over time (Supplementary Table 2). Blood (BLO) levels decreased in 3 samples, aligning with microscopy findings. Protein (PRO) decreased in sample 4, and Leukocytes (LEU) decreased in sample 2. Other components remained stable, except for single time-point fluctuations that returned to baseline.

## Discussion

Our study provides a comprehensive analysis of the temporal dynamics of human urine composition, utilizing both automated microscopy and dipstick analysis. We observed significant changes in several key urine constituents over a 6-h period. The notable increase in BACc count underscores the rapid proliferation of bacteria in urine samples stored at room temperature. Conversely, the marked decrease in RBC and PAT levels highlights the potential for degradation or lysis of these cellular elements over time. The fluctuating and inconsistent behavior of CRY and CRYu emphasizes the challenges in interpreting their presence in delayed urine samples. The relative stability of other components, such as pH, specific gravity, and various cellular elements such as WBCs, suggests their resilience to changes within the studied timeframe. However, it's important to acknowledge that we couldn't analyze nine components due to their high baseline %CV, indicating inherent variability that might obscure any time-dependent changes.

### Clinical implications of study findings

The clinical implications of these findings are substantial. In the context of hospitalized patients, where urine microscopy plays a pivotal role in diagnosing and managing kidney conditions [10], delays in sample processing can significantly impact the accuracy and interpretation of results. Hematuria, the presence of RBCs in urine, is a crucial indicator for various kidney diseases, including malignancy, glomerular disease, kidney stones, and urinary tract infections (UTIs). The observed decline in RBC levels over time could lead to missed diagnoses of hematuria, especially in cases where the initial count is close to the diagnostic threshold. For instance, in one patient, the RBC count dropped below the diagnostic threshold for microscopic hematuria (2 RBCs/HPF) after 3 h, potentially leading to an overlooked diagnosis [11].

Casts, particularly cellular casts like WBC and RBC casts, are significant markers for conditions such as acute interstitial nephritis (AIN) and vasculitis [12]. While we observed a modest decline in PAT over time, the IDEXX SediVue Dx®'s inability to differentiate between cellular and granular casts limits our understanding of their individual behavior [13]. This demonstrates the importance of manual microscopy for a comprehensive evaluation of casts in urine samples. The observed increase in bacterial count could lead to false-positive diagnoses of urinary tract infections, potentially triggering unnecessary antibiotic treatment with its associated risks and side effects.

### Constraints of automated microscopy

For a clinician, neutrophils and eosinophils are the types of WBCs that are most relevant to diagnosing conditions in disease. The IDEXX SediVue Dx ® does not differentiate types of WBCs and thus a clinician would not be able to determine the specific type of white blood cell present in a sample without manual microscopy. This limitation means that while the instrument can detect the presence of white blood cells in general, it cannot provide the more detailed information necessary to identify whether the cells are neutrophils, eosinophils, or another subtype. Similarly, the automated microscopy does not differentiate between granular and RTE cast. All the non-hyaline casts are labeled as pathological casts.

### Comparison of manual and automated microscopy

A key question is whether automated microscopy can truly match the performance of traditional manual microscopy. Previous studies have shown moderate agreement between the IDEXX SediVue Dx ® and manual microscopy for cast detection, with the SediVue demonstrating varying accuracy depending on the threshold used [6, 7]. These findings suggest that while automated microscopy offers advantages in terms of speed and standardization, manual microscopy might still be necessary for definitive diagnosis in certain cases.

### Methods to preserve urine in literature

Prior studies have shown that refrigeration of urine samples reduces component changes. Still, refrigeration can cause an increased count of crystals and amorphous urates that can affect the recognition of some components [14]. Although preservative tubes are available to maintain the sample, it is not common practice to use these tubes in a clinical setting, and they typically are only used in discovery studies [14]. Also, the expense associated with these tubes may be prohibitive for routine clinical practice. Additionally, chemical preservation can be used to keep the urine samples stable at room temperature, but the added cost and inconvenience make this method uncommon [15]. Prior studies have shown that WBCs stay preserved in acidic and hypertonic conditions [16].

### Strengths and limitations of the study

Our study is unique as it utilized reliable measurements from the state-of-the-art IDEXX SediVue Dx ® machine, providing high accuracy and reproducibility in microscopic examination. This instrument has not been used to analyze human urine samples; thus, there are no academic publications investigating its use in human urine at the time of the writing of this paper.

One of the limitations is that the investigation only spanned a relatively short time frame of 6 h, where longer delays in urine analysis might reveal further changes in the urine composition of other components. Another limitation was the small sample size as each urine sample has to be analyzed individually, limiting the number of samples that can be studied for test–retest changes at frequent intervals. Further studies on larger cohorts should be performed to confirm these findings. For example, there could be interaction of components such that these results may be different in urines with extremes of pH or in the presence of large amounts of protein or glucose.

## Conclusion

The study shows that a timely urinalysis is critical for accurate results since the delayed analysis can considerably change the makeup of the urine, thereby affecting clinical decisions and the management of patients [17]. Future studies should consider more extended time frames, diverse patient populations, and methods of sample handling (refrigeration or preservatives) and analysis techniques being more optimized. Additionally, a combination of automated and manual microscopy may offer the most comprehensive and accurate approach to urine analysis, leveraging the strengths of both techniques while mitigating their respective limitations.

## Supplementary Information

Supplementary Material 1.

## Data Availability

No datasets were generated or analysed during the current study.

## Abbreviations

UA: Urine Analysis
ICU: Intensive Care Unit
%CV: Percentage Coefficient of Variation
IRB: Institutional Review Board
BACc: Cocci-shaped bacteria
RBC: Red Blood Cells
PAT: Pathological Casts
WBC: White Blood Cells
CRY/CRYu: Crystals
BLO: Blood
PRO: Protein
LEU: Leukocytes
p/HPF: Particles Per High-Power Field
AIN: Acute Interstitial Nephritis
RTE: Renal Tubular Epithelial Casts
UTIs: Urinary Tract Infections
