# Supplementary material for: Changes in the composition of urine over six hours using urine dipstick analysis and automated microscopy
Source: BMC Nephrol. 2025 Jan 8;26:11. doi: 10.1186/s12882-024-03933-z (PMC11708174; doi:10.1186/s12882-024-03933-z)

**Supplementary Figures**

**Supplementary Table 1:** **Coefficient of Variations for Each Component**

|  | **Sample 1** | | | **Sample 3** | | | **Sample 5** | | |  |
| --- | --- | --- | --- | --- | --- | --- | --- | --- | --- | --- |
|  | **Mean** | **SD** | **%CV (±)** | **Mean** | **SD** | **%CV (±)** | **Mean** | **SD** | **%CV (±)** | **AVG %CV (±)** |
| RBC | 12.69 | 0.31 | 2.44 | 24.45 | 0.91 | 3.73 | 308.25 | 13.76 | 4.46 | 3.54 |
| WBC | 4.72 | 0.42 | 8.91 | 303.45 | 17.25 | 5.69 | 32.33 | 4.92 | 15.22 | 9.94 |
| CRY | 9.58 | 1.27 | 13.30 | 0.05 | 0.05 | 93.81 | 0.59 | 0.25 | 43.36 | 50.16 |
| .CaOxd^a^ | 0.00 | 0.00 | 0.00 | 0.00 | 0.00 | 0.00 | 0.00 | 0.00 | 0.00 | 0.00 |
| .TRI | 0.39 | 0.27 | 68.09 | 0.00 | 0.00 | 0.00 | 0.00 | 0.00 | 0.00 | 22.70 |
| .CRYu | 9.19 | 1.08 | 11.75 | 0.05 | 0.05 | 93.81 | 0.59 | 0.25 | 43.36 | 49.64 |
| .AMB^a^ | 0.00 | 0.00 | 0.00 | 0.00 | 0.00 | 0.00 | 0.00 | 0.00 | 0.00 | 0.00 |
| .BIL^a^ | 0.00 | 0.00 | 0.00 | 0.00 | 0.00 | 0.00 | 0.00 | 0.00 | 0.00 | 0.00 |
| HYA | 0.00 | 0.00 | 0.00 | 0.02 | 0.02 | 115.47 | 0.07 | 0.02 | 25.30 | 46.92 |
| PAT | 0.09 | 0.04 | 49.45 | 0.03 | 0.01 | 40.00 | 0.06 | 0.02 | 33.33 | 40.93 |
| NEC | 0.25 | 0.09 | 36.28 | 3.60 | 0.20 | 5.57 | 0.76 | 0.13 | 17.67 | 19.84 |
| EPI | 0.03 | 0.03 | 120.00 | 1.93 | 0.26 | 13.27 | 0.05 | 0.04 | 66.67 | 66.64 |
| YEA^a^ | 0.00 | 0.00 | 0.00 | 0.00 | 0.00 | 0.00 | 0.00 | 0.00 | 0.00 | 0.00 |
| BACr | 7.76 | 0.30 | 3.83 | 3.34 | 0.25 | 7.45 | 3.01 | 0.12 | 4.07 | 5.12 |
| BACc | 47.92 | 0.87 | 1.82 | 6.46 | 0.20 | 3.04 | 3.14 | 0.12 | 3.67 | 2.84 |
| .BACcs | 44.86 | 0.75 | 1.67 | 5.47 | 0.25 | 4.60 | 1.33 | 0.04 | 2.97 | 3.08 |
| .BACcc | 3.07 | 0.18 | 5.72 | 1.00 | 0.10 | 10.43 | 1.81 | 0.12 | 6.55 | 7.57 |
| MUC | 24.60 | 1.56 | 6.35 | 12.78 | 1.32 | 10.32 | 138.20 | 5.41 | 3.91 | 6.86 |
| SPRM | 0.03 | 0.04 | 121.46 | 0.00 | 0.00 | 0.00 | 0.00 | 0.00 | 0.00 | 40.49 |
| CRYd | 0.29 | 0.10 | 36.30 | 0.06 | 0.04 | 58.97 | 8.54 | 1.36 | 15.98 | 37.08 |

The components with a %CV of <20 are highlighted in grey. The Hour 6 timepoint is used for all three samples.

CV = coefficient of variation

SD = standard deviation

^a^ Were not observed in the sample that were tested.

**Supplementary Table 2: Summary of Trends in Dipstick Components**

| **GLU (mg/dL) (Glucose)** | | | | | |
| --- | --- | --- | --- | --- | --- |
|  | Hour 0 | Hour 1 | Hour 2 | Hour 4 | Hour 6 |
| Sample 1 | Negative | Negative | Negative | Negative | Negative |
| Sample 2 | 100 | 100 | Negative | Negative | 100 |
| Sample 3 | Negative | Negative | Negative | Negative | Negative |
| Sample 4 | Negative | Negative | Negative | Negative | Negative |
| Sample 5 | Negative | Negative | Negative | Negative | Negative |
| **BIL (Bilirubin)** | | | | | |
|  | Hour 0 | Hour 1 | Hour 2 | Hour 4 | Hour 6 |
| Sample 1 | Negative | Negative | Negative | Negative | Negative |
| Sample 2 | Large | Large | Large | Large | Large |
| Sample 3 | Negative | Negative | Negative | Negative | Negative |
| Sample 4 | Negative | Negative | Negative | Negative | Negative |
| Sample 5 | Small | Small | Small | Small | Small |
| **KET (mg/dL) (Ketone)** | | | | | |
|  | Hour 0 | Hour 1 | Hour 2 | Hour 4 | Hour 6 |
| Sample 1 | Negative | Negative | Negative | Negative | Negative |
| Sample 2 | Negative | Trace | Trace | Trace | Negative |
| Sample 3 | Negative | Negative | Negative | Negative | Negative |
| Sample 4 | Negative | Negative | Negative | Negative | Negative |
| Sample 5 | 15 | 15 | 15 | 15 | 15 |
| **SG (Specific Gravity)** | | | | | |
|  | Hour 0 | Hour 1 | Hour 2 | Hour 4 | Hour 6 |
| Sample 1 | 1.02 | 1.015 | 1.02 | 1.02 | 1.015 |
| Sample 2 | 1.02 | 1.02 | 1.02 | 1.02 | 1.02 |
| Sample 3 | 1.02 | 1.02 | 1.02 | 1.02 | 1.025 |
| Sample 4 | 1.02 | 1.02 | 1.03 | 1.025 | 1.02 |
| Sample 5 | 1.03 | 1.03 | 1.03 | 1.03 | 1.03 |
| **BLO (Blood)** | | | | | |
|  | Hour 0 | Hour 1 | Hour 2 | Hour 4 | Hour 6 |
| Sample 1 | Large | Large | Large | Moderate | Moderate |
| Sample 2 | Large | Large | Moderate | Moderate | Moderate |
| Sample 3 | Large | Large | Moderate | Moderate | Moderate |
| Sample 4 | Moderate | Moderate | Moderate | Moderate | Moderate |
| Sample 5 | Large | Large | Large | Large | Large |
| **pH** | | | | | |
|  | Hour 0 | Hour 1 | Hour 2 | Hour 4 | Hour 6 |
| Sample 1 | 5.5 | 5.5 | 6 | 5.5 | 5.5 |
| Sample 2 | 7 | 7 | 7 | 7 | 7 |
| Sample 3 | 6 | 6 | 6 | 6 | 6.5 |
| Sample 4 | 6 | 6 | 6 | 6 | 6.5 |
| Sample 5 | 7 | 7 | 6.5 | 7 | 6.5 |
| **PRO (mg/dL) (Protein)** | | | | | |
|  | Hour 0 | Hour 1 | Hour 2 | Hour 4 | Hour 6 |
| Sample 1 | 100 | 100 | 100 | 100 | 100 |
| Sample 2 | 300 | 300 | 300 | 300 | 300 |
| Sample 3 | 100 | 100 | 100 | 100 | 100 |
| Sample 4 | 300 | 100 | 100 | 100 | 100 |
| Sample 5 | 100 | 100 | 100 | 100 | 100 |
| **URO (E.U./dL) (Urobilinogen)** | | | | | |
|  | Hour 0 | Hour 1 | Hour 2 | Hour 4 | Hour 6 |
| Sample 1 | 1 | 1 | 1 | 1 | 1 |
| Sample 2 | 1 | 0.2 | 0.2 | 0.2 | 1 |
| Sample 3 | 0.2 | 0.2 | 0.2 | 0.2 | 0.2 |
| Sample 4 | 0.2 | 0.2 | 0.2 | 0.2 | 0.2 |
| Sample 5 | 1 | 1 | 1 | 1 | 1 |
| **NIT (Nitrite)** | | | | | |
|  | Hour 0 | Hour 1 | Hour 2 | Hour 4 | Hour 6 |
| Sample 1 | Negative | Negative | Negative | Negative | Negative |
| Sample 2 | Negative | Negative | Negative | Negative | Negative |
| Sample 3 | Negative | Negative | Negative | Negative | Negative |
| Sample 4 | Negative | Negative | Negative | Negative | Negative |
| Sample 5 | Negative | Negative | Negative | Negative | Negative |
| **LEU (Leukocyte)** | | | | | |
|  | Hour 0 | Hour 1 | Hour 2 | Hour 4 | Hour 6 |
| Sample 1 | Negative | Trace | Negative | Negative | Negative |
| Sample 2 | Trace | Trace | Negative | Negative | Negative |
| Sample 3 | Large | Large | Large | Large | Large |
| Sample 4 | Negative | Negative | Negative | Negative | Negative |
| Sample 5 | Negative | Negative | Negative | Negative | Negative |

**Supplementary Figure 1: Average Trends Over Time for Cocci-shaped Bacteria (BACc), Red Blood Cells (RBC), Pathological Casts (PAT), and Crystals (CRY/CRYu)**


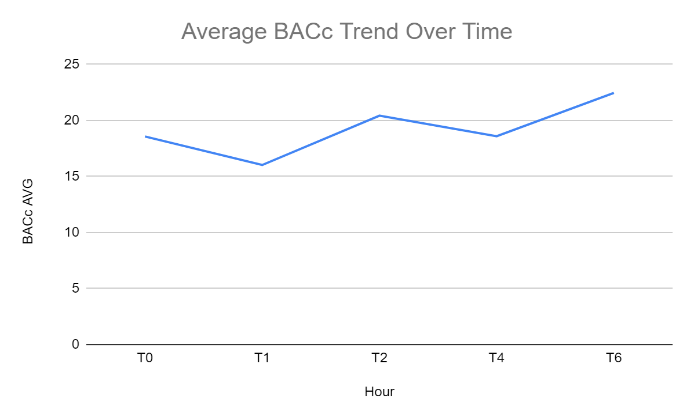

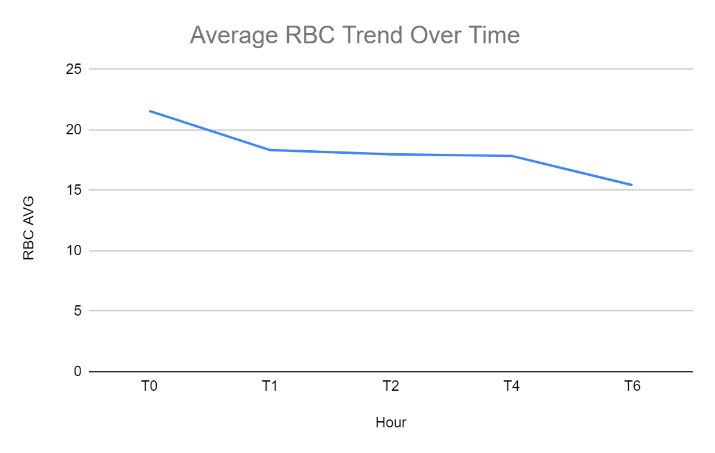


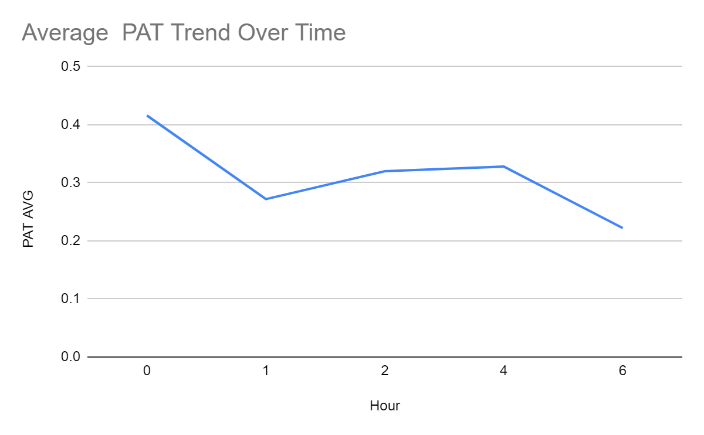

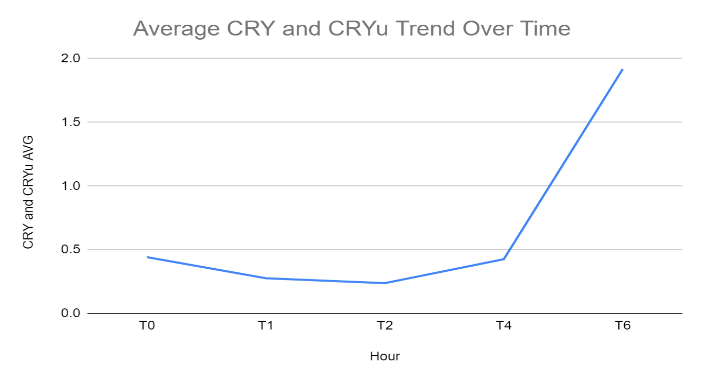

Supplement: Supplementary file 1 — Supplementary Material 1. [file 12882_2024_3933_MOESM1_ESM.docx]
